# Nitric oxide–an antidote to seed aging modifies *meta*-tyrosine content and expression of aging-linked genes in apple embryos

**DOI:** 10.3389/fpls.2022.929245

**Published:** 2022-08-30

**Authors:** Katarzyna Ciacka, Marcin Tyminski, Agnieszka Wal, Agnieszka Gniazdowska, Urszula Krasuska

**Affiliations:** Department of Plant Physiology, Institute of Biology, Warsaw University of Life Sciences, Warsaw, Poland

**Keywords:** apple seeds, Hsp, Lea, ROS, Saur, Tor, artificial aging

## Abstract

Short-term (3 h) treatment of embryos isolated from accelerated aged apple seeds (*Malus domestica* Borkh.) with nitric oxide (NO) partially reduced the effects of aging. The study aimed to investigate the impact of the short-term NO treatment of embryos isolated from apple seeds subjected to accelerated aging on the expression of genes potentially linked to the regulation of seed aging. Apple seeds were artificially aged for 7, 14, or 21 days. Then, the embryos were isolated from the seeds, treated with NO, and cultured for 48 h. Progression of seed aging was associated with the decreased transcript levels of most of the analyzed genes (*Lea1*, *Lea2a*, *Lea4*, *Hsp70b*, *Hsp20a*, *Hsp20b*, *ClpB1*, *ClpB4*, *Cpn60a*, *Cpn60b*, *Raptor*, and *Saur*). The role of NO in the mitigation of seed aging depended on the duration of the aging. After 7 and 14 days of seed aging, a decreased expression of genes potentially associated with the promotion of aging (*Tor*, *Raptor*, *Saur*) was noted. NO-dependent regulation of seed aging was associated with the stimulation of the expression of genes encoding chaperones and proteins involved in the repair of damaged proteins. After NO application, the greatest upregulation of *ClpB*, *Pimt* was noted in the embryos isolated from seeds subjected to 7-day long accelerated aging, *Hsp70b*, *Hsp70c*, and *Cpn* in the embryos of seeds aged for 14 days, and *Lea2a* in the embryos of seeds after 21 days of aging. We also demonstrated the increased *meta*-tyrosine concentration depending or in respect the progression of artificial aging, and the NO-induced increased phenylalanine content in seeds artificially aged for 21 days. In the NO-treated embryos of seeds aged for 7 and 21 days, the level of tyrosine was almost doubled compared to the aged tissue. Our data confirmed the usage of *meta*-tyrosine as a marker of seed aging and indicated that the increased *meta*-tyrosine/tyrosine ratio could be related to the loss of seed viability.

## Introduction

Seed aging is a natural process of great impact from an ecological, agronomical, and economical point of view, thus understanding its regulation is still of great interest and importance. The beneficial effect of NO on the maintenance of seed vigor during aging was recently reviewed by Ciacka et al. (2020). The application of NO or its donors before implementation of the aging protocol for elm (*Ulmus pumila* L.) seeds activated the protection mechanisms that prevented the reduction of vigor in aged seeds (He et al., 2018). NO treatment of oat (*Avena sativa* L.) seeds after accelerated aging also improved their quality (Mao et al., 2018).

Apple (*Malus domestica* Borkh.) seeds belong to the *orthodox* type, and seeds of this type are rather insensitive to environmental factors, and, generally, age slowly. Apple seeds are characterized by a deep embryonic dormancy, which is removed by 3 months-long cold stratifications (4–5°C) (Lewak, 2011). This process is accompanied by an increased emission of NO from the embryonic axes (Dȩbska et al., 2013), and in a laboratory may be replaced by a short-term (3 h) fumigation of isolated embryos with NO (Gniazdowska et al., 2010). Embryos isolated from cold stratified apple seeds germinate fast and developing seedlings are of typical features (Lewak, 2011; Dȩbska et al., 2013). In contrast, warm stratification of apple seeds (25°C) did not lead to dormancy removal. The NO emission from the axes of warm stratified seeds was lower in comparison to the NO emission from the axes isolated from cold stratified seeds (Dȩbska et al., 2013). Conditions of warm stratification are similar to those described in accelerated aging protocols. Our previously published data have shown that keeping dormant apple seeds in wet sand at 33°C for 14 and 21 days may be successfully used to achieve material for studies of seed artificial aging (Ciacka et al., 2022). We have proven that NO may be regarded as a remedy against oxidative damages in the apple seeds undergoing accelerated aging (Ciacka et al., 2022).

Oxidative damages due to a cellular redox imbalance are at the center of the free radical theory of aging (Corbineau, 2012; Kurek et al., 2019), thus, a balanced reactive oxygen species (ROS) metabolism is crucial for the preservation of seed vigor. In the embryos of aged apple seeds, a stimulation of the total antioxidant capacity after NO fumigation was demonstrated. It was accompanied by increased transcript levels of genes encoding enzymatic antioxidants, resulting in the protection of the embryonic tissue against overaccumulation of ROS (Ciacka et al., 2022). In addition, we have analyzed in detail many aspects of ROS action in seeds during artificial aging and its changes in response to NO treatment (Ciacka et al., 2022). Thus, in the current research, we have decided to measure the content of *meta*-tyrosine (*m*-Tyr), the structural Tyr isomer, which is a product of the oxidation of phenylalanine (Phe). It may serve as a marker of oxidative stress (Ipson and Fisher, 2016; Tyminski et al., 2021 and references therein). One of the toxic actions of *m*-Tyr in plants is its impact on ROS and reactive nitrogen species (RNS) metabolism (Krasuska et al., 2017; Andrzejczak et al., 2018). The higher concentration of *m*-Tyr is typical for patients suffering from neurodegenerative diseases and diseases associated with oxidative stress and/or aging (Tyminski et al., 2021 and references therein). Consequently, we suspect that the prolonged artificial aging of apple seeds could result in the accumulation of *m*-Tyr in the embryonic axes, and this effect could be altered by the treatment with NO.

Seed quality and viability may be estimated by the analysis of the expression of genes encoding proteins belonging to Heat shock proteins (Hsp) and Late embryogenesis abundant proteins (Lea) (Smolikova et al., 2021). Higher levels of Hsp seem to be necessary for the extension of the seed’s life span and the improvement of seedlings’ resistance to a variety of stresses (Kaur et al., 2015; Thakur, 2020). Hsp is a molecular chaperone, involved in protein folding and stability maintenance, as well as in protein protection against oxidative damage (Job et al., 2005). Arabidopsis seeds with an enhanced accumulation of Hsp were characterized by an improved tolerance to aging (Prieto-Dapena et al., 2006). The casein lytic proteinase/heat shock proteins 100 (Clp/Hsp100) are chaperones that may alter the formation of protein aggregates in an ATP-depending manner (Lee et al., 2006). Remodeling properties of Clp are related to the changes in the activity of protein complexes, unfolding protein for its degradation, or facilitating the refolding of protein aggregates. In plants, one of the best-studied proteins from this family is cytosolic ClpB. The ClpB/Hsp100 expression is regulated by heat stress and developmental signals (Lee et al., 2006). Rice (*Oryza sativa* L.), wheat (*Triticum aestivum* L.), maize (*Zea mays* L.), and Chinese mustard [*Brassica juncea* L.(Czern.)] seeds constitutively accumulate high levels of ClpB (Mishra and Grover, 2016).

Chaperonins (Cpn, known also as Hsp60) regulate protein folding, assembly, and translocation (Ishikawa et al., 2003). Hsp60 proteins interact with histidine kinase and participate in phytohormonal signaling and resistance to stresses (Nagaraju et al., 2021 and references therein).

Lea proteins are known as the key elements in sustaining seed viability (Smolikova et al., 2021 and references therein). A decreased content of Lea2 proteins was noticed as beech (*Fagus sylvatica* L.) seeds storage was prolonged (Kalemba and Pukacka, 2008). After 8 years of seed storage, a 2-fold decrease in the content of Lea2 in embryonic axes, in comparison to that in seeds stored for only 1 year, was observed. In contrast, the content of Lea in beech seeds increased during seed maturation in correlation to tissue dehydration (Kalemba and Pukacka, 2014). Lea is stable in a wide range of temperatures. During seed dehydration, these proteins act as chaperons, stabilizing the structure of other proteins and cell membranes, in case of protein damages that promote their refolding (Smolikova et al., 2021 and references therein).

Natural or accelerated aging of organisms or organs, e.g., seeds result in a conversion of L-aspartyl or asparaginyl residues to abnormal isoaspartyl (isoAsp) residues (Mudgett et al., 1997; Sano et al., 2016), and lead to the loss of proteins’ function. L-Isoaspartyl *O*-methyltransferase (Pimt) repairs these abnormal residues. The overexpression of genes encoding Pimt led to the decrease in isoAsp residue accumulation in seed proteins and increased seed longevity, while the decreased Pimt level resulted in the loss of seed viability (Ogeì et al., 2008; Rajjou and Debeaujon, 2008 and references therein; Verma et al., 2013). Thus, Pimt-mediated protein repair is important for the regulation of seeds’ lifespan.

The target of rapamycin–Tor is an evolutionary conserved Ser/Thr protein kinase playing the important role in the control of cell proliferation and growth in Eukaryotes (Xiong and Sheen, 2014; Kravchenko et al., 2015). In plants, Tor signaling is engaged in the regulation of embryogenesis, meristem activation, root development and growth, flowering, and lifespan determination. Its action in seeds is under the control of phytohormones involved in the regulation of seed germination (Salem et al., 2017; Salem and Giavalisco, 2019). In plants, Tor interacts with two proteins: regulatory associated partner of Tor (Raptor) and lethal with sec-thirteen protein 8 (Lst8), forming the TorC1 (Moreau et al., 2012; Kravchenko et al., 2015; Salem et al., 2017; Salem and Giavalisco, 2019). Tor is suggested to control ribosomal biogenesis, polysome accumulation, and various protein translational processes, as well as rRNA transcription (Ben-Shem et al., 2011; Xiong and Sheen, 2014), especially important during prolonged seed imbibition, and early seedling growth. In addition, in seeds, TOR regulates β-oxidation of stored lipids and oils (Xiong et al., 2013; Xiong and Sheen, 2014).

Small auxin-up RNA (*Saur*) is the largest family of early auxin-responsive genes in higher plants. In Arabidopsis, its number is above 80 (Zhang et al., 2021). *Saur* genes have been shown to play roles in diverse processes of plant growth, development, and stress responses (high temperature, drought, or salt). Overexpression of *AtSaur49* was linked to the promotion of leaf senescence (Zhang et al., 2021 and references therein).

As indicated by many authors, seed aging is accompanied by changes in the content of proteins mentioned above (Rajjou et al., 2008). These proteins are regarded as markers of seed longevity, because they are involved in protein folding, stabilizing, and repairing protein structure (Lea, Hsp, Pimt), and are potentially related to autophagy regulation (Tor, Raptor, and Saur). Thus, the expression of genes: *Lea*, *Hsp*, *Pimt*, *Tor*, *Raptor*, and *Saur* were at the center of our interest related to NO-dependent restoration of apple seeds viability after 7 to 21 days of accelerated aging. Furthermore, we analyzed the content of *m*-Tyr, its proteinogenic analog—Phe, and the total concentration of its structural isomer—Tyr. Determination of *m*-Tyr in the apple embryos may confirm the universal harmfulness of this non-proteinogenic amino acid in Eukaryotes correlated to the destabilization of ROS metabolism during aging.

## Materials and methods

### Plant material

As a biological material apple (*Malus domestica* Borkh. cv. Antonówka) seeds subjected to the accelerated aging protocol according to Ciacka et al. (2022) were used. The ripened apple fruits were obtained from the WIKPOL fruit producer (Da̧browa, Poland). Seeds after isolation from apple fruits were dried and stored in dark glass containers at 5°C to preserve dormancy.

Dry apple seeds (100 seeds) were placed in a Petri dish (Ø 15 cm) filled with 300 g of sterile quartz sand (sand volume 180 ml), and moistured with 110 ml of sterile, distilled water. Accelerated aging was performed at 33°C (in Nahita incubator, 636 Plus) for 7, 14, and 21 days in 4 independent replications (each replication consisted of 8–10 Petri dishes). After aging, seeds were removed from the sand, rinsed with water, and the embryos were isolated. The embryos, treated with NO, were shortly fumigated with vapors of a solution of acidified nitrite [14.5 mM NaNO_2_ (Chempur 117926907), 0.2 M HCl] for exactly 3 h as described by Ciacka et al. (2022). After NO fumigation, the embryos were rinsed in distilled water. The non-treated embryos (the control, embryos isolated from seeds only subjected to accelerated aging) and the embryos isolated from seeds subjected to aging and shortly treated with NO (NO treatment) were placed in glass Petri dishes on filter paper moistened with distilled water for 48 h in a growth chamber (Sanyo MLR-35OH) in 25/20°C day/night, 12/12 h photoperiod, and with the light intensity of 100 μmol PAR m^–2^ s^–1^. After 48 h of the culture, the embryonic axes (50 embryonic axes corresponding to approximately 50 mg FW per sample) were isolated and used for experiments.

The experimental model was presented in Ciacka et al. (2022).

### Determination of amino acids concentration

Determination of Tyr, Phe, and *m-*Tyr concentration in the embryonic axes isolated from the embryos of aged apple seeds (the control) and the embryos treated with NO (NO treatment) after isolation from seeds subjected to accelerated aging was performed as described by Du et al. (2004) with modifications. The experiment was conducted with HPLC-grade chemicals. For samples derivatization, 1 mM of the 6-aminoquinolyl-hydroxysuccinimidyl carbamate (AQC) (Cayman Chemical 30877) dissolved in acetonitrile (HPLC grade, Poch 102644151) was used.

The embryonic axes (50 mg) were homogenized in 100% methanol (HPLC grade, Poch 621995156) (0.5 mL) with the addition of 2 μl of the internal standard 0.1 mM 5-methyl-DL-tryptophan (Sigma M0534). Homogenized samples were vigorously mixed and centrifuged at 20,000 *g* for 10 min at room temperature. Supernatants were collected and dried under vacuum (Eppendorf concentrator plus) to remove methanol. The obtained samples were dissolved in 100 μl of acetonitrile (HPLC grade).

Before separation, 20 μl of samples were mixed well with 70 μl of 0.2 M borate buffer pH 8.8 (sodium tetraborate, Sigma B9876; boric acid, Sigma 31146) and 10 μl of 1 mM AQC, and incubated for 10 min at 55°C in darkness.

After derivatization, 20 μl of the sample was injected onto the Bionacom Velocity LPH C18 column (4.6 × 150; 3 mm) that was held in a thermostat set at 30°C. For peak detection, the FP-2020/2025 Intelligent Fluorescence Detector (JASCO) was used (E_x_ 250 nm, E_m_ 395 nm). The mobile phase flow rate was set at 1 ml min^–1^. As mobile phase A, 0.15 M acetate buffer pH 6.15 (sodium acetate, Chempur 118056403; acetic acid, Poch BA8760114) with 0.033% (v/v) triethylamine (TEA, Sigma T0886) was used. Acetonitrile was used as mobile phase B.

The following gradient program was used for samples separation: 0–10 min 84–80% A, 10–21 min 80–70% A, 21–25 min 70–60% A, 25–27 min 60–50% A, 27–30 min 50–20% A, 30–31 min 20–10% A, 31–32 min 10–30% A, 32–35 min 30–60% A, and 37–37 min 60–84% A.

As standards, L-Tyr (J&K 185061), L-Phe (Sigma P17008), and *m*-Tyr (J&K 263747) were used. Standard amino acids were mixed with 0.2 M borate buffer pH 8.8, derivatized with 1 mM AQC, and separated under the conditions described above.

The analysis was performed using three biological repetitions, in three technical replications. The concentration of amino acids was presented as pmol g^–1^ FW.

### Analysis of genes expression

Total RNA was isolated from approximately 50 mg of embryonic axes, using RNAzol^®^ RT, according to Andryka-Dudek et al. (2019). After treatment with DNAse I (Thermo Scientific EN0523), total RNA (200 ng) was used for cDNA synthesis. The cDNA was obtained using the RevertAid First Strand cDNA Synthesis Kit (Thermo Scientific #K1622) in a total volume of 10 μl. The finished product was diluted 3.5 times. A qPCR was carried out in a Bio-Rad CFX Connect™ Real-Time PCR Detection System. The iTaq™ universal SYBR^®^ Green supermix (Bio-Rad #172–5124) was used for the reaction in a total volume of 12 μl (6 μl PCR supermix, 1 μl cDNA, 1 μl primer, and 4 μl sterile H_2_O). Specific primers were designed based on nucleotide sequences available in the National Center for Biotechnological Information (NCBI) and the Genome Database for Rosaceae^1^ (Supplementary Table 1). As an effect of using the BLASTP algorithm (NCBI), the sequences of the encoded proteins were aligned with Arabidopsis proteins. The name of the corresponding protein in Arabidopsis and the reference number in the Uniprot database can be found in Supplementary Table 1.

The expression levels were normalized using two reference genes and calculated using the method described by Vandesompele et al. (2002) and Hellemans et al. (2007). The experiments were done in three biological replicates, in three technical repetitions.

### Statistics

**FIGURE 1:**
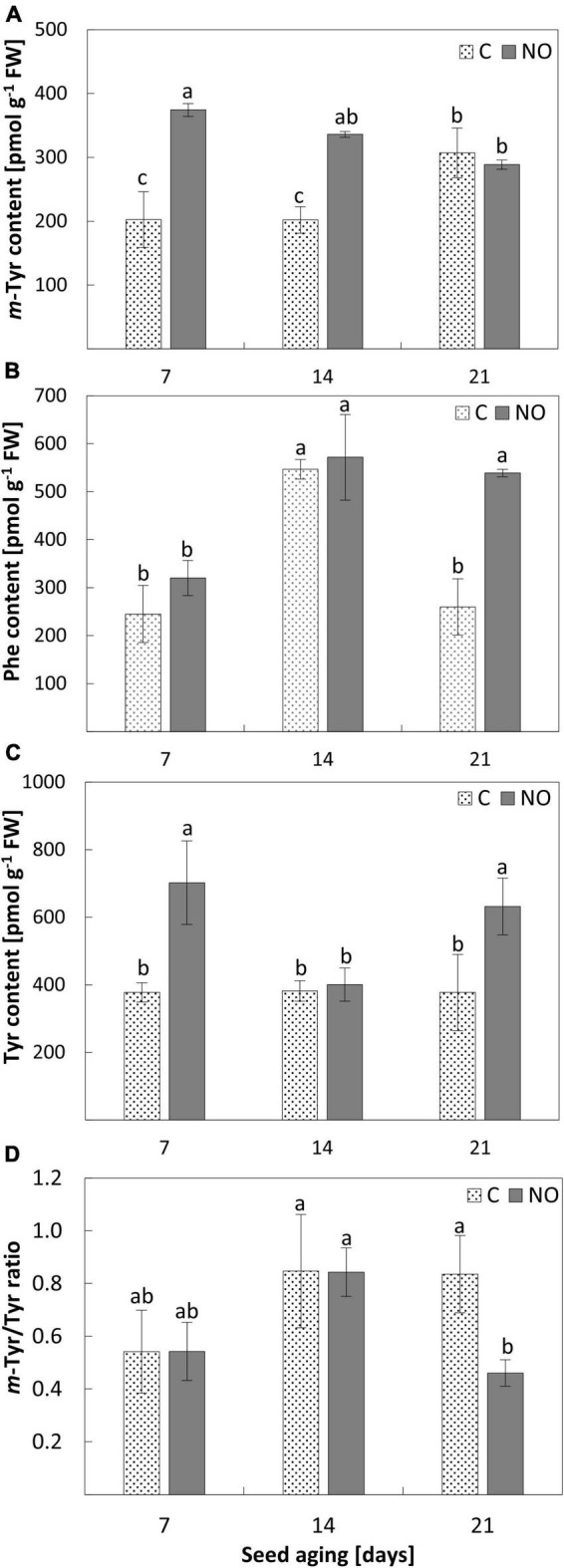
The content of *m*-Tyr **(A)**, Phe **(B)**, Tyr **(C)**, and the *m*-Tyr/Tyr ratio **(D)** in the axes of apple seeds subjected to accelerated aging for 7, 14, and 21 days (C) and in the axes of apple embryos fumigated with NO after seeds accelerated aging (NO). Values are average ± SD of 3 independent repetitions. Letters indicate the significant differences determined by Duncan’s test (*P* < 0.05).

**FIGURE 2:**
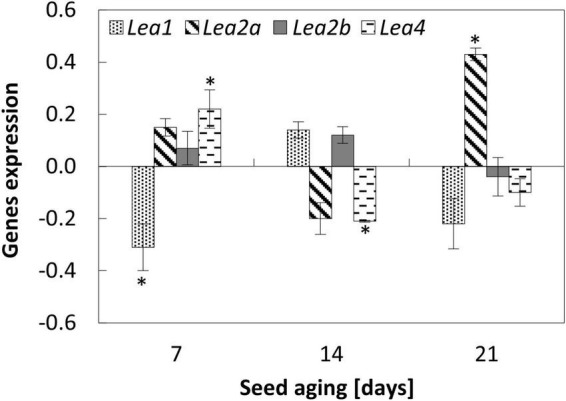
NO-dependent changes in the expression of *Lea1*, *Lea2a*, *Lea2b*, and *Lea4* in the embryonic axes of apple seeds subjected to accelerated aging for 7, 14, and 21 days. Values are average ± SD of 3 repetitions. Asterisks indicate significant differences between the treated sample compared to the control sample, obtained by the Student’s *t-*test (*P* < 0.05).

**FIGURE 3:**
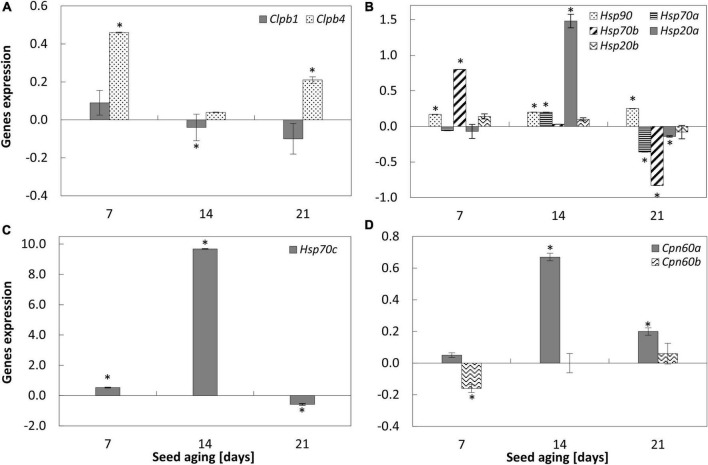
NO-induced modifications in the expression of *ClpB1* and *ClpB4* **(A)**, *Hsp90*, *Hsp70a*, *Hsp70b*, *Hsp20a*, *Hsp20b* **(B)**, *Hsp70c* **(C)**, *Cpn60a* and *Cpn60b* **(D)** in the embryonic axes of apple seeds subjected to accelerated aging for 7, 14, and 21 days. Values are average ± SD of 3 repetitions. Asterisks indicate significant differences between the treated sample compared to the control sample, obtained by the Student’s *t*-test (*P* < 0.05).

**FIGURE 4:**
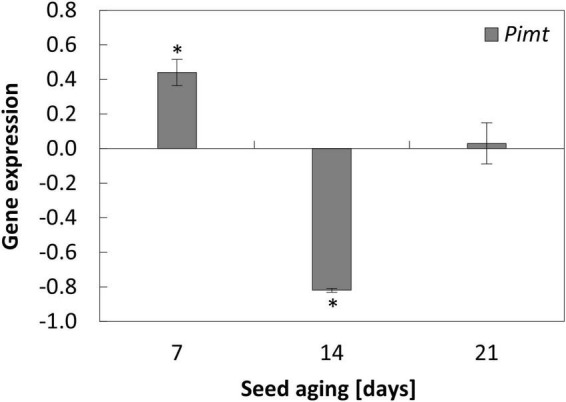
NO-dependent alterations in the expression of *Pimt* in the embryonic axes of apple seeds subjected to accelerated aging for 7, 14, and 21 days. Values are average ± SD of 3 repetitions. Asterisks indicate significant differences between the treated sample compared to the control sample, obtained by the Student’s *t*-test (*P* < 0.05).

**FIGURE 5:**
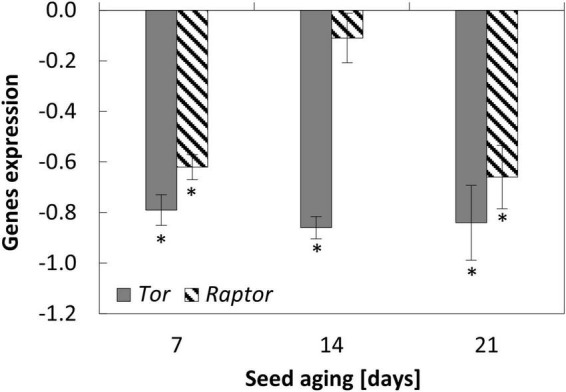
NO-dependent alterations in the expression of *Tor* and *Raptor* in the embryonic axes of apple seeds subjected to accelerated aging for 7, 14, and 21 days. Values are average ± SD of 3 repetitions. Asterisks indicate significant differences between the treated sample compared to the control sample, obtained by the Student’s *t*-test (*P* < 0.05).

**FIGURE 6:**
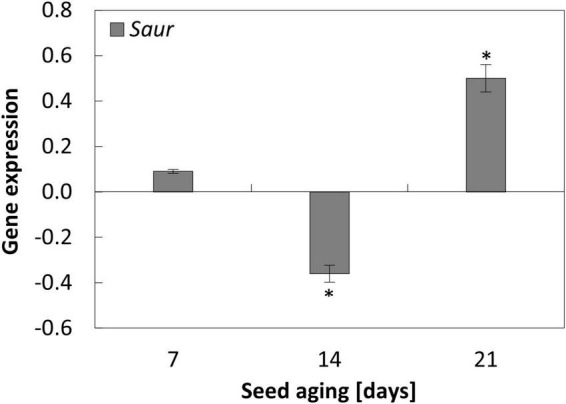
NO-dependent changes in the expression of *Saur* in the embryonic axes of apple seeds subjected to accelerated aging for 7, 14, and 21 days. Values are average ± SD of 3 repetitions. Asterisks indicate significant differences between the treated sample compared to the control sample, obtained by the Student’s *t*-test (*P* < 0.05).

The data were analyzed using the Statistica13 Software. All data were obtained from at least three independent experiments with at least three repetitions each and presented as mean values ± SD. For gene expression analysis, the significant differences were evaluated using Student’s *t*-test (Figures 2–6), or after one-way ANOVA, homogenous groups were evaluated using Tukey’s test (Supplementary Figure 2). For the results of *m*-Tyr, Phe, and Tyr content and *m*-Tyr-Tyr ratio Figure 1, two-way ANOVA was used to determine the statistically significant differences between the means. The homogeneous groups were obtained by Duncan’s test. In the supplement, the results of the content of amino acids in the axes of embryos after NO treatment expressed as a percentage of the control (the embryos of aged seeds) were analyzed using Student’s *t*-test (Supplementary Figure 1).

## Results

### Seed aging increased the *m*-Tyr level in the embryonic axes and nitric oxide modified the content of *m-*Tyr, Phe, Tyr, and *m-*Tyr/Tyr ratios in the embryonic axes of aged seeds

After 7 days of apple seeds’ accelerated aging, the *m*-Tyr concentration was approximately 200 pmol g^–1^ FW in the embryonic axes and did not change as aging lasted until 14 days. Treatment of these embryos (after seeds aging for 7 and 14 days) with NO resulted in an increase in the level of *m*-Tyr by 85 and 65%, respectively. Prolongation of seeds aging (until 21 days) increased the content of *m*-Tyr by approximately 50% compared to 7 and 14 days aged seeds. Fumigation of the embryos isolated from 21 days aged seeds with NO did not change the *m*-Tyr level in the axes (Figure 1A).

After 7 days of seed aging, the content of Phe was around 300 pmol g^–1^ FW, regardless of NO application (Figure 1B). The level of this amino acid increased by 80% in the axes of apple seeds subjected to accelerated aging for 14 days and the value of this parameter did not change after the embryos’ treatment with NO. After 21 days of seed aging, the content of Phe was similar to that observed in the embryonic axes of seeds aged for the shortest period (7 days). Fumigation of these embryos with NO increased the content of Phe in the axes more than twice, to the level observed in the axes of seeds aged for 14 days (Figure 1B).

The total content of Tyr in the embryonic axes of aged seeds remained constant irrespectively of the duration of aging (Figure 1C). NO application increased the level of this amino acid in seeds aged for the shortest (7 days) and the longest (21 days) period by approximately 85 and 65%, respectively. In the axes of embryos isolated from seeds aged for 14 days, fumigation with NO did not influence Tyr content (Figure 1B).

The highest value of the *m*-Tyr/Tyr ratio was characteristic for the axes of embryos isolated from seeds aged for 14 days (regardless of the embryos’ treatment with NO) and 21 days (non-treated with NO) (Figure 1D). The embryonic axes of seeds aged for 7 days differed in the mean value of the ratio in comparison to the tissue aged for 14 and 21 days, but this difference was not statistically significant. NO fumigation did not change the value of the *m*-Tyr/Tyr ratio in the axes of embryos of seeds aged for a week or two. While, after 21 days of seeds aging, NO application resulted in a 2-fold decrease in the value of the *m*-Tyr/Tyr ratio compared to the control (Figure 1D).

### Nitric oxide modified the expression of *Lea* in the embryonic axes of aged seeds

Accelerated aging of apple seeds resulted in the decreased transcript levels of *Lea1*, *Lea2a*, and *Lea4* in the embryonic axes. Only for the transcript level of *Lea2b* fluctuations with the minimum on the 14th day of aging was noticed (Supplementary Figure 2).

After 7 days of accelerated aging, the embryos’ fumigation with NO increased the expression of gene encoding Lea4 (Figure 2). In the axes of these embryos, the decrease in *Lea1* and no changes in *Lea2a* and *Lea2b* expression levels were noticed. After 14 days of seed artificial aging, NO application altered only the expression of *Lea4*, which was downregulated. In the axes of apple embryos isolated from seeds aged for 21 days, NO treatment led to the upregulation of *Lea2a* (Figure 2), while the expression of other genes did not differ.

### Nitric oxide upregulated the expression of *Hsp* in the embryonic axes of aged seeds

Accelerated aging of apple seeds resulted in a gradual decrease in *Clpb1* and *Clpb4* transcript levels in the embryonic axes (Supplementary Figure 2).

Nitric oxide fumigation of apple embryos isolated from aged seeds changed the expression of one of the two studied genes encoding proteins belonging to the Clp/Hsp100 family. The increase in the transcript levels of *ClpB4* was noticed in the embryonic axes of seeds aged 7 and 21 days (Figure 3A).

Progression of accelerated aging of apple seeds resulted in the decrease in the transcript levels of most of the analyzed genes encoding Hsp proteins. The downregulation of *Hsp70b*, *Hsp20a*, *Hsp20a*, and *Hsp20b* was observed (Supplementary Figure 2). Transcript levels of *Hsp90* and *Hsp70a* were stable irrespectively of the duration of the aging.

Nitric oxide treatment of the embryos isolated from seeds aged for 7 days resulted in the increase in the expression of *Hsp90*, *Hsp70b*, and *Hsp70c* (Figures 3B,C), while the transcript levels of *Hsp70a*, *Hsp20a*, and *Hsp20b* did not change. After 14 days of seed aging, NO upregulated the expression of most of these genes, especially *Hsp70c* (Figures 3B,C). In the axes of seeds subjected to accelerated aging for 21 days and after being exposed to NO, a decrease in the expression of *Hsp70a*,*b*,*c*, and *Hsp20a* was observed. The transcript level of *Hsp90* was upregulated, while the expression of *Hsp20b* did not differ (Figure 3B).

Accelerated aging of apple seeds decreased the transcript levels of both analyzed genes (*Cpn60a* and *Cpn60b*), known also as *Hsp60*, encoding chaperonins (Supplementary Figure 2). Although the greatest reduction in the transcript level was observed for 14-day-long aged seeds (Supplementary Figure 2). As a result of NO treatment, *Cpn60a* was upregulated in the axes of apple embryos of seeds subjected to accelerated aging for 14 and 21 days, while the expression of *Cpn60b* decreased after 7 days of seed aging (Figure 3D).

### Nitric oxide modified the expression of *Pimt* as seed aging lasted up to 14 days

Accelerated aging of apple seeds resulted in the fluctuation of *Pimt* transcript levels in the embryonic axes. Its maximum was noticed after 14 days of seed aging, while prolongation of the aging resulted in the downregulation of the gene, which expression was even lower than in the axes of 7-day-aged seeds (Supplementary Figure 2).

The NO-dependent alterations in the transcript level of *Pimt* were characteristic for the axes of apple embryos isolated from seeds aged for 7 and 14 days (Figure 4). When the duration of the aging procedure was shorter, the expression of *Pimt* was upregulated. After 14 days of aging, NO application decreased the transcript level of *Pimt* (Figure 4).

### Nitric oxide downregulated the expression of genes encoding elements of Tor complex in the embryonic axes of aged seeds

Accelerated aging of the seeds for 14 and 21 days decreased the transcript level of *Raptor*, while the changes of *Tor* transcript level exhibited the fluctuation with the minimum at the 14th day (Supplementary Figure 2).

Nitric oxide application resulted in the decrease in the expression of *Tor* and *Raptor* in the axes of apple seeds after 7 and 21 days of aging. After 14 days of aging, NO treatment led to the decrease in the transcript level of *Tor*, while the expression of *Raptor* did not differ (Figure 5).

### Nitric oxide changed the expression of *Saur* when seed aging lasted for at least 14 days

In the axes of seeds subjected to accelerated aging protocol, a decrease in the expression of *Saur* was observed. Its transcript level was similar after 14 and 21 days of aging (Supplementary Figure 2).

The changes in the expression of *Saur* after NO treatment were noticed when seeds were aged for 14 and 21 days. In the axes of apple seeds aged for 2 weeks, the transcript level was downregulated, while after 21 days, the increase in its expression was observed after embryos’ fumigation with NO (Figure 6).

## Discussion

**FIGURE 7:**
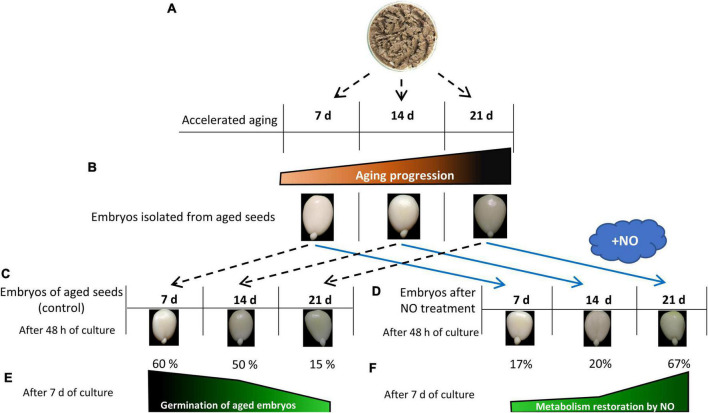
The diagram describes the basic features of the biological material and the scheme of the experiment. Apple seeds were subjected to accelerated aging for 7, 14, or 21 days **(A)**. After seed aging embryos were isolated **(B)**. The typical embryos isolated from aged seeds [control **(C)**] and the typical NO-treated embryos after 48 h of culture are shown panel **(D)**. Progression of seed aging was linked to the gradual decrease in germination rate of the embryos after 7 and 14 days, and a drop-down in the germination of the embryos as the aging lasted for 21 days **(E)**. NO acted as a remedy against aging by the increase in germination rate of the embryos isolated from aged seeds [metabolism restoration **(F)**]. Metabolism restoration (%) was calculated based on the germination rate of the embryos after NO fumigation according to data presented by Ciacka et al. (2022). Embryos isolated from aged seeds and after NO treatment were viable (as indicated by the TTC test) as described by Ciacka et al. (2022).

**FIGURE 8:**
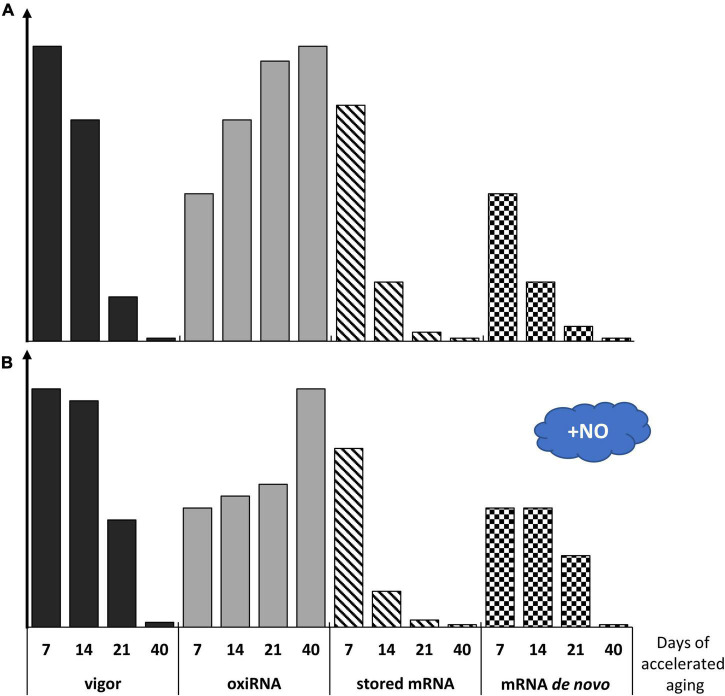
The figure describes the vigor of apple embryos in dependence on the progression of seed accelerated aging concerning the changes in the level of the oxidized RNA (oxiRNA), stored mRNA, and newly synthesized RNA (mRNA *de novo*) in the control embryos, non-treated with NO **(A)**, and the embryos shortly fumigated with NO **(B)**. The height of the bars filled with color (vigor-black and oxiRNA-gray) is proposed according to data by Ciacka et al. (2022). The height of the bars filled with sloping lines–stored mRNA, is estimated according to Zhao et al. (2020a; 2020b). The height of the checkered bars–mRNA *de novo* is hypothetical.

Recently, the conditions for the accelerated aging of apple seeds have been established (Ciacka et al., 2022). Embryos of apple seeds placed at 33°C and 65% humidity for more than 14 days exhibited the visible symptoms of aging, while after 40 days, more than 70% of seeds died (Ciacka et al., 2022; Figures 7, 8). One week of accelerated aging resulted in the partial removal of the dormancy of apple seeds. It can be assumed, that during 7-day aging, the apple seeds undergo imbibition, but these conditions do not provide the overall metabolic changes required for the entire dormancy breakage. These embryos need a long period of imbibition (the culture) to accomplish germination. After 7 days of accelerated aging, they germinated in 60% after 1 week of the culture (Ciacka et al., 2022; Figure 7). Based on previous results, we regarded this stage as the early phase of apple seeds’ artificial aging. After 48 h of the culture under conditions favorable for germination, the embryonic axes of seeds aged for 7, 14, and 21 days do not elongate nor bent gravitropically (Figure 7). In our experiments, we used the axes of apple embryos isolated from aged seeds and shortly treated with NO. To detect the long-term effect of NO application, it was necessary to culture embryos for 48 h under favorable conditions (Figure 7).

The role of NO in delaying the negative effects of seed aging is partially linked to the modulation of ROS metabolism (He et al., 2018; Mao et al., 2018; Ciacka et al., 2022). Stable molecular markers pointing to the progression of seed aging are needed to determine seed quality. In human tissue, *m*-Tyr is regarded as such an indicator, because its accumulation corresponds to the induction of oxidative stress (Ipson and Fisher, 2016). *m*-Tyr content increased as accelerated aging was prolonged and in the embryonic axes of apple seeds subjected to 21 days of aging was approximately twice higher than in the embryonic axes of seeds aged for 7 or 14 days. It corresponded to the increase in malondialdehyde content (Ciacka et al., 2022) and pointed at the oxidative stress, induced during seed aging, thus, confirming the universality of *m*-Tyr as a marker of aging in living organisms. Supplementation of young tomato seedlings with *m*-Tyr (50 μM) resulted in an increased ROS/RNS level in the roots (Krasuska et al., 2017; Andrzejczak et al., 2018). Thus, the elevated level of this non-proteinogenic amino acid may, in part, explain the increased content of ROS in aged embryos (Dȩbska et al., 2013; Ciacka et al., 2022). The *m-*Tyr is an antimetabolite of Phe, and both amino acids are competitors for tRNA*^Phe^* (Bertin et al., 2007; Rodgers et al., 2017). Phe at high concentration protects the tissue against the toxic action of *m*-Tyr, lowering its incorporation into protein structures. The recovery effects of Phe were demonstrated for tomato (Andrzejczak et al., 2018) and Arabidopsis (Zer et al., 2020) plants supplemented with *m*-Tyr. Thus, the fluctuation in the levels of Phe in the embryos of aged apple seeds (non-treated with NO) indicates that one of the explanations for the cellular deterioration observed after 21-day artificial aging could be the disruption of the Phe/*m*-Tyr level, favoring *m*-Tyr accumulation. The embryos isolated from seeds, aged for 21 days and treated with NO, were characterized by a similar *m*-Tyr level to that in the control seeds but with a 2-fold higher content of Phe. Moreover, in these embryos, the ratio of *m*-Tyr to Tyr was lower, mostly due to the increased content of the total Tyr. The high free Tyr level induced by NO may act *via* the intracellular amino acids’ isoform competition, mitigating advanced aging processes. After 7 and 14 days of accelerated aging of apple seeds in the axes of NO-treated embryos, the *m*-Tyr content was higher compared to the aged seeds, while Phe was at a similar level (Supplementary Figure 1). Therefore, it can be proposed that NO, by the stimulation of peroxynitrite (ONOO^–^) formation, leads to Phe oxidation, resulting in the accumulation of *m*-Tyr. Surprisingly, after NO treatment, the decrease in the Phe content in the axes of 7 and 14 days aged seeds was not detected. It may be due to the putative increase in protein degradation, stimulated by NO (Krasuska et al., 2014), especially that the increased Tyr content after 7 days of aging was observed. The increased degradation of proteins during advanced aging may also be the reason for *m*-Tyr release from oxidized proteins. Moreover, it was suggested that the transcriptional coordination of Phe, and not the Tyr biosynthesis with its upstream and downstream pathways, likely supports the preferred synthesis of Phe over Tyr in plants (Schenck and Maeda, 2018); therefore, NO action could restore this regularity. The direct or non-direct impact of NO on Phe metabolism in the aspect of *m*-Tyr accumulation needs further investigation.

According to previous data, 7 days of accelerated aging of apple seeds did not induce any significant, visible damages (Ciacka et al., 2022; Figure 7). Additionally, NO fumigation of the embryos isolated from seeds aged for 7 days has slightly accelerated the germination rate and enhanced the percentage of the properly grown seedlings (Ciacka et al., 2022; Figure 7). In the axes of these embryos cultured for 48 h, only a few investigated genes were upregulated (*Lea4*, *ClpB4*, *Hsp90*, *Hsp70b*, and *Pimt*). Proteins encoded by these genes are responsible for repair processes and protein stabilization, which could be necessary for the correction of damages, which take place during dehydration at the seed maturation stage and rehydration in imbibition.

The impact of NO on seed longevity maintenance could be explained by different mechanisms of its action, depending on the duration of accelerated aging and the biochemical state of the seeds (Figure 8). This assumption is based on the abundance of the transcripts encoding enzymatic antioxidants after 21 days of seed aging (Ciacka et al., 2022). In addition, the duration of apple seed accelerated aging treatment and experimental time points used in our study were selected to match the pattern of seed viability decrease vs. time described by Walters et al. (2010). Seed aging is linked to a sigmoidal dependence between viability and storage time. The unspecified duration of seed viability (the early aging phase of no apparent changes) is followed by the rapid viability loss—a mortality phase. Furthermore, seed longevity depends on metabolism (the degree of oxidation of cellular molecules and accumulation of oxidants), strictly linked to seed moisture (Walters et al., 2010) and temperature. The imbibition at 33°C from 7 to 40 days differentiates the degree of apple embryo tissue hydration and physiological activity. So, metabolically, embryos isolated after each time point should be considered separately and detected alterations should not be viewed linearly.

The asymptomatic phase of seed aging (corresponding probably to 7 days of apple seeds aging) is hard to determine if based only on seed germination tests (Zhao et al., 2020a,b). It has been proposed that stored mRNA (the amount and quality) may serve as an indicator of the progression of seed aging (Zhao et al., 2020a,b). The authors using Arabidopsis seeds indicated that stored mRNA degraded at a constant rate over the aging time. However, there is no information about the metabolic use of stored mRNA in artificially aged seeds as they are subjected to imbibition. The degradation of stored mRNA may also be linked to the rate of its oxidation.

Since our work concerns alterations in the transcript levels of the genes related to seed aging, we propose a hypothetical model describing changes in vigor, stored mRNA level, newly synthesized mRNA (mRNA *de novo*), and oxidized RNA (oxiRNA) in the embryonic axes of apple seeds subjected to accelerated aging for 7–40 days (Figure 8A). We suggest that until the 7th day of accelerated aging, embryos remind in the asymptomatic phase, and thus, are characterized by only minor changes in metabolism and quality. A decline in embryo vigor observed after 14 days of accelerated aging corresponds to rapid mortality and leads to death at the time point of 40 days (Figure 8A).

Continuing the investigation of the molecular action of NO during seed aging, we suggest that NO prolongs the asymptomatic phase and shallows the mortality phase (Figure 8B), which is related to the alterations in the expression of genes potentially involved in the regulation of seed longevity.

Lea proteins influence seed longevity by participation in the formation of the glassy state. The accelerated aging conditions promote the loss of the glassy state of the embryo and enhance seed deterioration (Hundertmark et al., 2011). Lea proteins accumulate during seed maturation, as well as in vegetative tissues in response to water deficit (Liu et al., 2011). In plants, there is a large number of genes encoding Lea (Kalemba and Pukacka, 2008; Leprince et al., 2016). Based on the sequence similarity and properties, Lea is divided into 7 groups (Leprince et al., 2016). Lea of the 2nd group is known as dehydrins, whereas proteins from the 4th and 5th groups are possibly involved in the membrane stability maintenance. During water loss, Lea proteins act as chaperons, prevent protein aggregation, and modulate ROS formation, acting as scavengers of transitional metal ions, e.g., Fe^3+^ (Alsheikh et al., 2003; Liu et al., 2011). After synthesis during seed maturation, Lea is transported to different cellular organelles and membranes to protect and stabilize molecules. During seed imbibition, most of the mRNA of *Lea* is gradually degraded (Kalemba and Pukacka, 2007 and references therein). The protective role of Lea during seed maturation is well-known, whereas there is no evidence concerning their role in the relief of the effect of degenerative processes related to seed aging. The seeds obtained from Arabidopsis lines with a significant reduction in *Lea* expression stored at 35°C and 75% relative humidity (the glassy state of the cytoplasm is likely no longer exist) exerted the reduced viability (Hundertmark et al., 2011). The authors proposed that some Lea proteins play a protective role even in seeds that lost their glassy state, thus, the decrease in the level of dehydrins affects seed storability. In our experiment, the transcript levels of *Lea1*, *Lea2a*, and *Lea4* decreased in response to seed aging, which corresponds well to the low germination rate (Figure 7). It is also proposed that Lea exhibits different functions depending on the cellular water status (Leprince et al., 2016). In beech seeds that have been stored at −10°C and 8–9% moisture content, an increased level of Lea was noted, especially in the embryonic axes (Kalemba and Pukacka, 2008). NO-fumigated apple embryos isolated from seeds aged for 21 days were characterized by the highest abundance of *Lea2a*, and it was not observed in the axes of seeds subjected to the shorter period of accelerated aging. During seed maturation, the accumulation of stored mRNAs or long-lived mRNAs (encoding, e.g., 12S seed storage proteins and dehydrins) was detected. These mRNAs were used in protein translation during the early phases of germination (Rajjou et al., 2004; Sano et al., 2020) and probably in germinating embryos isolated from the seeds in the asymptomatic phase of aging (Figure 8). If we assume that 48 h of the embryo’s culture corresponds to the early germination stage, especially after 7 and 14 days of accelerated aging, NO may promote the translation of Lea proteins (Lea1 or Lea4) from stored mRNA. After NO treatment, transcript levels of these genes decreased. The prolongation of apple seeds’ accelerated aging favors ROS formation (Ciacka et al., 2022; Figure 8). Thus, the NO-induced expression of the specific *Lea* could be a protective mechanism against ROS overaccumulation. Such Lea may serve as free radicals modulators by binding metal ions (Liu et al., 2011).

Besides Lea, Hsp is another protein engaged in plant stress responses. Depending on their molecular weight, these proteins are divided into six classes: Hsp100 (Clp), Hsp90, Hsp70, Hsp60 (Cpn), Hsp40, and small Hsp (sHsp) (Kalemba and Pukacka, 2007; Kumar et al., 2020). They are accumulated at the late stage of seed maturation, and during germination, their abundance decreases constantly. The decreased transcript levels of genes encoding Hsp and ClpB proteins were observed as apple seed aging was in progress (Supplementary Figure 2). The protective role of Hsp is related to their chaperone function. They participate in the resolubilization of protein aggregates and assist in the folding of nascent polypeptides (Mayer and Bukau, 2005; Kumar et al., 2020). Hsp is accumulated in plant tissue under high temperatures. The increase in sHsp level was found in the embryos of dry maize seeds artificially aged at 50°C (Xin et al., 2011) and in the embryos of dry wheat and barley seeds subjected to 40°C (Dell’Aquila et al., 1998). Our experiments have demonstrated the diverse impact of NO-treatment on the level of various *Hsp* transcripts, depending on the progression of seeds aging. In the axes of embryos isolated from seeds aged for 14 days, as the result of NO treatment connected with the increase in the embryos’ vigor, the upregulation of *Hsp70a*, *Hsp70c*, and *Hsp20a* was detected; whereas after 21 days of aging, the treatment of the embryos with NO led to a decrease in the expression of these genes, as well as *Hsp70b.* In turn, for these embryos, a slight increase in the level of *ClpB4* was noted. ClpB proteins, known as Hsp100, are involved in the maintenance of protein structure. Probably, they took part in the dissolution of cytosolic or nuclear protein aggregates formed during heat stress (Hong and Vierling, 2001 and references therein). The study using the Arabidopsis mutant characterized by the complete absence of the Hsp101 showed that plant growth, as well as seed germination, was not affected by the lack of this protein (Hong and Vierling, 2001). Authors suggested that Hsp101 activity is not critical in the absence of stress, although the biosynthesis of Hsp101 is regulated during seed development and the proteins are stored in dry seeds. In contrast, the mutation related to the reduction of the Hsp101 level led to the decrease in the thermotolerance of germinating Arabidopsis seeds. This fact emphasized the importance of Hsp101 to seed biology during stress conditions, probably due to their chaperone, and the putative translational regulatory functions (Hong and Vierling, 2001). The role of Hsp101 in the germination of Arabidopsis seeds subjected to heat stress was also proven by Queitsch et al. (2000).

The mode of action of NO in the regulation of viability of apple embryos isolated from seeds aged for 14 and 21 days was associated with the increase in the expression of both *Hsp90* and *Cpn60a*. However, the NO-dependent upregulation of *Cpn60a* in the axes of the seeds aged for 21 days, in comparison to the control, was not so noticeable as for the seeds aged for 14 days. The viability of NO-treated embryos of 21-day aged seeds is not so high as embryos of 14-day aged seeds (Figure 7). The germinability of NO-treated embryos after 21 days of aging was only 50% of that after 14 days of aging (Ciacka et al., 2022). As aging was prolonged up to 21 days, the NO-dependent increase in the expression of *ClpB* was accompanied by the decrease in the expression of *Hsp70a* and *Hsp70b*. Therefore, we may assume that the mechanism of regulation of protein structure by Hsp is insufficient in this tissue in comparison to the NO-treated embryos isolated from seeds aged for 14 days, especially since the disaggregation of proteins may require the cooperation of Hsp100, Hsp70, and Hsp40 homologs (Queitsch et al., 2000 and references therein). The enhancement of the level of transcripts encoding protein “protectors” (Hsp100 or Hsp60), after NO application, especially in the earlier stage of aging, may prevent the putative accumulation of protein aggregates that may occur as a result of oxidative stress (Ciacka et al., 2020).

The aging of chickpea (*Cicer arietinum* L.) seeds increased the level of isoaspartyl residues in proteins and decreased the Pimt activity (Verma et al., 2013). The enhancement of vigor and longevity of chickpea seed were connected with the increased expression of *CaPimt* (Verma et al., 2013). Similarly, the increased expression of *Pimt1* was important in the preservation of vigor and extended longevity of Arabidopsis seeds, subjected to the controlled deterioration treatment (40°C, 82% relative humidity) (Ogeì et al., 2008). In this experiment, the highest transcript level of *Pimt* was characteristic for the axes of 14-day aged apple seeds. It corresponds to the relatively high germination rate of these embryos (Figure 7), suggesting also the activation of some repair mechanisms at this stage of aging progression. The most profitable effect of NO action on apple embryo viability was confirmed by the higher level of *Pimt* in 7-day aged seeds when the aging-induced degradation of cellular components is not so advanced. The decrease in expression of *Pimt* was characteristic of the axes of NO-treated embryos of seeds aged for 14 days, which can be explained by the drop-down of stored mRNA as the aging process was in progression (Figure 8). The lowest transcript level of *Pimt* was observed in the axes of 21-day-aged seeds. In these embryos, NO did not alter the gene expression, which may be reflected in the worst germinability.

In the last 5 years, the number of information regarding Tor activity in plant physiology has increased. In plants, Tor is involved in the control of mRNA translation, protein synthesis, and life span determination (Quilichini et al., 2019). The treatment of wheat (*Triticum aestivum* L.) seeds with rapamycin or torin1 (the inhibitors of Tor activity) resulted in the inhibition of germination under favorable conditions (Smailov et al., 2020). The authors proposed that the Tor signaling pathway is involved in the GA-dependent synthesis of α-amylase and following seed germination. As was demonstrated for Arabidopsis, Raptor, the essential element of Tor machinery, also regulates the undisturbed seed germination. The delay in Arabidopsis seed germination was observed for mutants with a loss of function in *raptor1B* (Salem et al., 2018). The authors indicated that Raptor1B is a positive regulator of seed germination (examined under favorable conditions), and is involved in the maintenance of hormonal and nutritional balance. Delayed germination of *raptor1b* seeds (the knockout mutant) was accompanied by an increase in the level of phytohormones: ABA and auxin (Salem and Giavalisco, 2019). Inhibition of seed germination in *Raptor* mutants corresponded to the lowering transcript level of *Raptor* in the axes of aged apple seeds, in which viability and germination were reduced (Figure 7).

Regardless of the duration of apple seeds aging, the NO-dependent downregulation in *Tor* and *Raptor* (except for seeds aged for 14 days) expression was observed. As mentioned above, in seeds, the role of Tor is related to the regulation of seed germination, especially at the level of the transition from heterotrophic to photoautotrophic growth (McCready et al., 2020). Regardless of the duration of seed aging and the embryos’ treatment with NO, after 2 days of the culture, apple embryos do not differ visually (Figure 7). As mentioned in the first part of the discussion, these embryos are characterized by short, not bent embryonic axes, which means they are not at the stage of the transition into seedlings. Therefore, we were not surprised by the decrease in *Tor* and *Raptor* expression after embryos’ treatment with NO. The upregulation of *Lea4*, *ClpB4*, *Hsp90*, *Hsp70b*, or *Pimt* seems to be the priority because reparation of some damages that occurred during, e.g., seed rehydration is required. Thus, the removal of damages is a critical point to follow the completion of germination and seedling development. Going on further, NO may even prevent the accumulation of *Tor* and *Raptor* to lower proliferation activity and minimize the damage. Whether the *Tor* and *Raptor* transcripts belong to stored mRNA and NO regulates its utility, needs additional experiments.

In plants, Tor acts as a negative regulator of autophagy, the process which is necessary to recycle cytoplasmic contents under stress conditions or during senescence/aging (Liu and Bassham, 2010). An increasing number of studies have proven the selective autophagic-dependent degradation of various cell components (Su et al., 2020 and references therein), which may prevent early aging under stress conditions. In the axes of NO-treated apple embryos, the reduced levels of *Tor* (after 14 and 21 days of aging) and *Raptor* (after 21 days of aging) may promote autophagy events. Autophagy can be induced by various pathways, one of which is related to ROS (Pérez-Pérez et al., 2012). Alteration in Tor activity is also dependent on the cellular ROS level. The decrease in Tor activity in Arabidopsis seedlings under oxidative stress conditions was noted (Ahn et al., 2019). However, the studies conducted on Arabidopsis mutants with the downregulation of *Tor* by RNA interference have shown that NADPH oxidase (involved in ROS – O_2_^∙–^ generation) inhibitors did not block the constitutive autophagy in this plant (Liu and Bassham, 2010). Previous research on aged apple seeds has shown the NO-dependent stimulation of the expression of genes encoding NADPH oxidase homologs (especially in the axes of embryos isolated from seeds aged for 21 days) (Ciacka et al., 2022). Therefore, we may assume that the autophagy in this tissue can be regulated *via* various pathways depending on the progression of tissue aging, but further research is needed to confirm this statement.

The Tor-dependent regulation of autophagy in aged apple seeds may also be controlled by auxin. After 14 days, the mechanism of NO action in the mitigation of the seed aging effect is also connected with the decrease in *Saur* (the auxin response gene) expression. *Saur* analyzed in our experiment, encodes Saur36 protein in Arabidopsis. According to the Uniprot database, this protein is responsible for the regulation of seed germination (Supplementary Table 1). Moreover, Saur36 functions as a positive regulator of leaf senescence and may mediate auxin-induced leaf senescence (Supplementary Table 1). After 14 days of aging of apple seeds, the lower expression of *Saur* in the axes of NO-treated embryos, compared to the control, may indicate the decrease in auxin level in this tissue. It has been shown that auxin triggers Tor activation (Schepetilnikov et al., 2013). It suggests that this phytohormone is connected with the negative regulation of autophagy. It depends on the type of stress affecting plant tissue (Pu et al., 2017). In the experiment with Arabidopsis mutant with *Tor* overexpression, the addition of auxin–1-naphthaleneacetic acid (NAA) inhibited autophagy during nutrient deficiency, salt, or osmotic stress, but not, e.g., during oxidative stress. Moreover, NAA treatment was unable to block autophagy induced by a Tor inhibitor or by a mutation in the *raptor1B* (the knockout line), indicating that auxin is upstream of Tor in the regulation of autophagy (Pu et al., 2017). It means that autophagy can be controlled *via* Tor-dependent and -independent pathways in plant tissue, and the Tor signaling pathway requires auxin as its regulator. Therefore, in the apple embryos of aged seeds, NO-treatment did not only impair the expression of genes encoding elements of the Tor complex, but it could also affect Tor activity through the regulation of auxin level. The direct impact of NO on Tor activity (*via* nitration/*S*-nitrosation of TorC1 components) or indirect [*via* nutrient availability (Pu et al., 2017)] needs further investigation. The possible regulation of autophagy by NO and the extension of seed longevity *via* TorC1 activity modulation also needs to be experimentally demonstrated. Taking all the above together, in non-stress conditions, proteins encoded by *Tor* and *Raptor* promote seed germination and seedling development, while when the seeds are aged, it is necessary to temporarily lower the level of TorC1 elements to recycle cell components and/or repair the oxidative damage.

## Conclusion

Prolonged conditions of accelerated aging resulted in visible symptoms of apple seeds aging and a drastic decrease in their germinability. Our current study pointed to the universal usage of *m*-Tyr as a marker of cellular aging, although it should be mentioned that more studies on Phe metabolism are necessary to fill the knowledge on the role of these amino acids in the regulation of aging processes. We have demonstrated that NO may be used as a remedy against some deteriorations occurring in seeds during artificial aging. The beneficial effect of NO on the restoration of proper metabolism of seeds was observed predominantly in the tissue, in which no severe damages were induced by prolonged high moisture and high temperature. The upregulation of genes encoding some Lea or Hsp after NO treatment of embryos of aged seeds explained the involvement of this RNS in the preservation of seed longevity, which is strengthened by the downregulation of *Tor* and *Raptor*. Alterations in the transcript levels of the genes indicate a putative important role of NO in the regulation of aging processes in seeds, but they cannot be directly related to the activity of the protein. These data point to the requirement for further analysis of the level and activity of individual proteins - the products of the analyzed genes. The regulatory action of NO in seed aging may be linked to its reactivity with RNA (nitration and oxidation). Recently, we have demonstrated that NO fumigation of apple embryos isolated from seeds subjected to accelerated aging has modified RNA nitration and oxidation level (Ciacka et al., 2022), although we have no data on which specific transcripts have been modified. It cannot be ruled out that the downregulation of some of the genes analyzed in this experiment was due to such NO-direct regulation. The differences in transcript levels after NO application in the embryos after 14 vs. 21 days of aging and 14 vs. 7 days of aging may be related to the variant use of stored mRNA (degradation vs. metabolic use), as well as dissimilar rate of newly synthesized mRNA. We propose that NO application may stimulate the use of stored mRNA, lower the oxidation level of RNA, and stimulate synthesis *de novo* of mRNA (Figure 8).

## Data availability statement

The datasets presented in this study can be found in online repositories. The names of the repository/repositories and accession number(s) can be found below: https://www.ncbi.nlm.nih.gov/geo/query/acc.cgi?acc=GSE207591, GSE207591.

## Author contributions

KC: conceptualization, methodology, investigation, data curation, writing – original draft preparation, and writing – review and editing. UK: conceptualization, writing – review and editing, and funding. MT: investigation and writing—editing. AW: investigation. AG: writing – review and editing. All authors have read and approved the final version of the manuscript.

## Footnotes

1
https://www.rosaceae.org/
