# Supplementary material for: Nitric oxide–an antidote to seed aging modifies meta-tyrosine content and expression of aging-linked genes in apple embryos
Source: Front Plant Sci. 2022 Aug 30;13:929245. doi: 10.3389/fpls.2022.929245 (PMC9468924; doi:10.3389/fpls.2022.929245)
Supplement: Supplementary file 1 [file Table_1.DOCX]

Supplementary Table 1. List of primers. The name of the corresponding protein in Arabidopsis and its reference no. in the Uniprot database were marked using Asterisk (*)

| **Gene symbol** | **Primer sequence 5’-3’** | **Encoded protein** | **Gene ID (GDR, NCBI)** |
| --- | --- | --- | --- |
| ***Tor*** | F:ATCTGGTGGAGCAGCTTTGC  R:TCGTTATACCGTTCTGCATCAC | Target of rapamycin/ *Serine/threonine-protein kinase TOR  Q9FR53 | MD10G1112000  MDP0000177219  MDP0000258439 |
| ***Raptor*** | F:TGAATGCTGTTGTGGATTGG  R:GCTACAATCTGATGAAGAAGG | TOR binding protein/ *Regulatory-associated protein of TOR 1  Q93YQ1 | MDP0000061332  MDP0000061330  MDP0000209431 |
| ***Saur*** | F:CATGTCAAGGCTGCTCACCTGG  R:CCAACCGCTTGTAACCCGAC | Small auxin up-regulated RNA/ *Auxin-responsive protein SAUR36  O22150 | MDP0000807487 |
| ***Pimt*** | F:GATGGTGGATCATTTGAAGAG  R:CTATCAATAGTCTCCATCACTTCAG | Protein l-isoaspartyl methyltransferase/ *Protein-L-isoaspartate O-methyltransferase 1  Q42539 | MDP0000314934  MDP0000299692 |
| ***Lea1*** | F:CACCAGATGTCCGCACTTC  R:ACCCGTGTTGGTCCCAATA | Late-embryogenesis abundant protein/ *Late embryogenesis abundant protein 46  Q9FG31 | MDP0000850643  MDP0000187047 |
| ***Lea2a*** | F:TCCACTACTCCTCCACC  R:ATATCCACAGCGGCGTC | Late-embryogenesis abundant protein/ *WHy domain-containing protein  A0A384KR80 | MDP0000808291  MDP0000385140 |
| ***Lea2b*** | F:GGATCAAGTACGGCGAGTCCAG  R:ACGACTTGGCGGACAGTGT | Late-embryogenesis abundant protein/ *LEA_2 domain-containing protein  A0A178VWF6 | MDP0000281672  MDP0000552607  MDP0000282151 |
| ***Lea4*** | F:GTCGATGATGAAGGCAGTGAC  R:GCCCTTCTCTTGAACCTGACT | Late-embryogenesis abundant protein/ *Late embryogenesis abundant protein ECP63  Q9SKP0 | MDP0000183836  MDP0000183375  MDP0000305723 |
| ***ClpB1*** | F:GAGACGGTTGGACCAGAACA  R:CCACTACTCTCTTATGCAACCT | Caseinolytic peptidase B protein homolog/ *Chaperone protein ClpB1  P42730 | MDP0000755970  MDP0000217508  MDP0000303015  MDP0000637620 |
| ***ClpB4*** | F:GCAGTTCTCTCAGACAGATACATTAC  R:TCGGTCTACCTCATCCAACT | Caseinolytic peptidase B protein homolog/ *Chaperone protein ClpB4, mitochondrial Q8VYJ7 | MDP0000242288  MDP0000304292  MDP0000304623 |
| ***Hsp90*** | F:TTCCAGGCCGAGATCAAC  R:GAAAGATCTCCTTGTTGCTGTA | Heat shock proteins 90 kDa/ *Heat shock protein 90-1  P27323 | MDP0000254260  MDP0000303430 |
| ***Hsp70a*** | F:TTGTCGGGTCAGGGTCTAG  R:AGCATTGCACATTGAAGAGCG | Heat shock protein 70 kDa/ *Heat shock 70 kDa protein 16  Q9SAB1 | MDP0000682297  MDP0000295388  MDP0000277804  MDP0000279175  MDP0000779762 |
| ***Hsp70b*** | F:ATCACCAACGACAAGGGCA  R:CTTTGCCTCCACCTTCTTCT | Heat shock protein 70 kDa/ *Heat shock 70 kDa protein 1  P22953 | MDP0000322220 |
| ***Hsp70c*** | F:CAATGATGCTCAGAGACAGG  R:ACCACCAAGATCAAATACAGC | Heat shock protein 70 kDa/ *Heat shock 70 kDa protein 10, mitochondrial  Q9LDZ0 | MDP0000416692 |
| ***Cpn60a*** | F:ACATCTGAAGAAATAGCTCAGG  R:CATTCCTTCAACGACCTCCAA | Chaperonin 60 kDa/ *Chaperonin CPN60, mitochondrial  P29197 | MDP0000859313  MDP0000235765 |
| ***Cpn60b*** | F:GCAGAGCATTGATGATAAGCAC  R:GTGTTCCCATCAGCAACAGTA | Chaperonin 60 kDa/ *Chaperonin CPN60-like 2, mitochondrial  Q93ZM7 | MDP0000185591 |
| ***Hsp20a*** | F:GAGAGGAAGAGGGAGCAGGA  R:AGCCGAAACCTCCTCGTGAAC | Heat shock protein 20 kDa/ *17.6 kDa class I heat shock protein 3  P13853 | MDP0000412799 |
| ***Hsp20b*** | F:TCCAGGTGGAGGACGACAA  R:CACAAACTTCCTCATGAACTTGC | Heat shock protein 20 kDa/ *17.6 kDa class II heat shock protein  P29830 | MDP0000294594  MDP0000700383  MDP0000418416 |
| ***Sar1*** | F: TTGATTTGGGCGGGCATCAGATTG  R: TCATCAGAGAGGAGAGCATCCAGC | Small GTP-binding protein | MD04G1194800 |
| ***Pdi*** | F: TGCTGTACACAGCCAACGAT  R: CATCTTTAGCGGCGTTATCCTTG | Protein disulfide isomerase | XM_008344461.2 |
