# Supplementary material for: Nitric oxide–an antidote to seed aging modifies meta-tyrosine content and expression of aging-linked genes in apple embryos
Source: Front Plant Sci. 2022 Aug 30;13:929245. doi: 10.3389/fpls.2022.929245 (PMC9468924; doi:10.3389/fpls.2022.929245)
Supplement: Supplementary file 2 [file Data_Sheet_1.PDF]

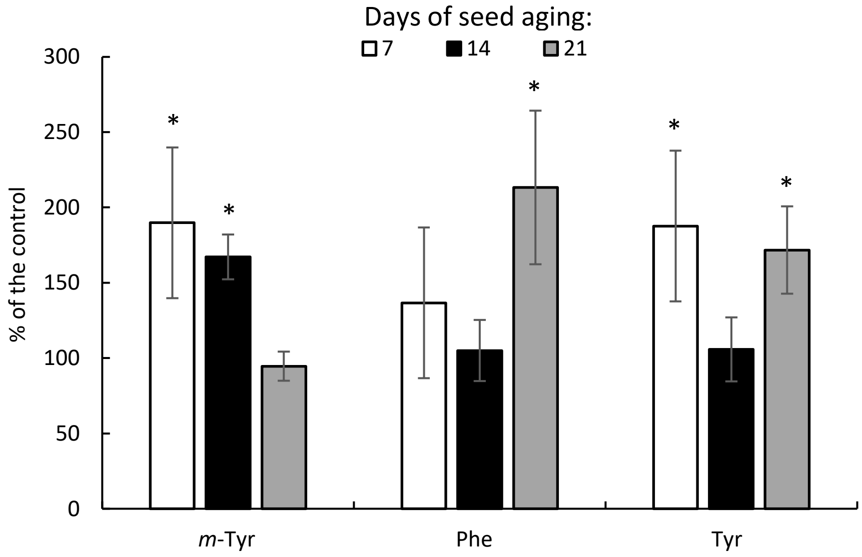

Supplemantary Figure 1. Changes in the content of *m*-Tyr, Phe and Tyr in the axes of embryos after NO treatment expressed as a percentage of the control (content of the amino acids in the axes of aged seeds). Values are average ± SD of 3 repetitions. Asterisks indicate significant differences determined by Student's t-test ($P<0.05$).
