# Supplementary material for: Nitric oxide–an antidote to seed aging modifies meta-tyrosine content and expression of aging-linked genes in apple embryos
Source: Front Plant Sci. 2022 Aug 30;13:929245. doi: 10.3389/fpls.2022.929245 (PMC9468924; doi:10.3389/fpls.2022.929245)
Supplement: Supplementary file 3 [file Data_Sheet_2.PDF]

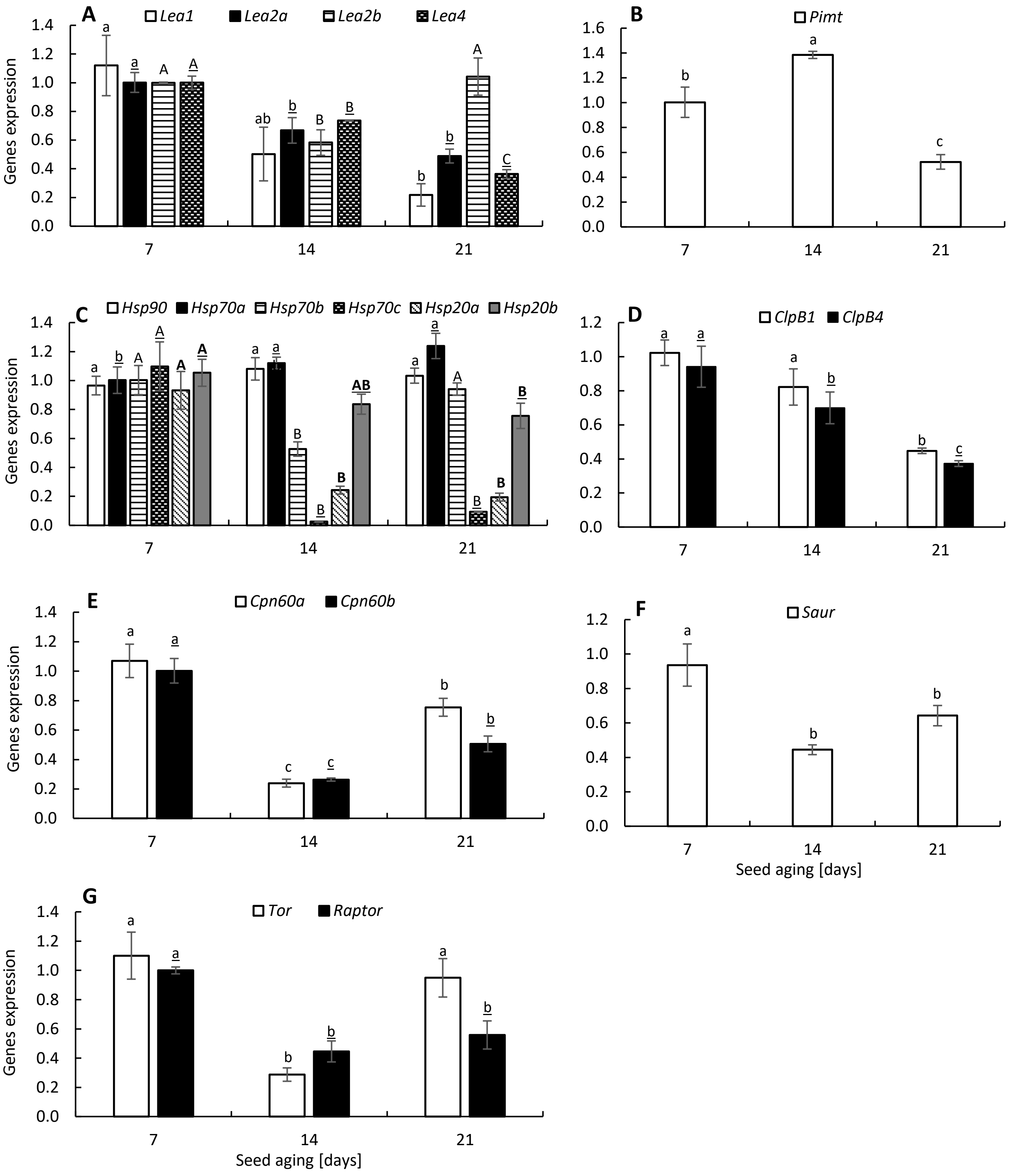

Supplemantary Figure 2. Changes in the expression of *Lea1*, *Lea2a*, *Lea2b*, *Lea4* (**A**) *Pimt* (**B**) *Hsp90*, *Hsp70a*, *Hsp70b*, *Hsp70c*, *Hsp20a*, *Hsp20b* (**C**), *ClpB1* and *ClpB4* (***D***), *Cpn60a* and *Cpn60b* (**E**) *Saur* (**F**) *Tor* and *Raptor* (**G**) in the embryonic axes of apple seeds subjected to accelerated aging for 7, 14, and 21 days. Values are average ± SD of 3 repetitions. After one-way ANOVA, homogenous groups were evaluated using the Tukey's test (P<0.05) and signed as a-c (*Lea1*, *Pimt*, *Hsp90*, *ClpB1*, *Cpn60a*, *Saur*, *Tor*), a-c (*Lea2a*, *Hsp70a*, *ClpB4*, *Cpn60b*, *Raptor*), A-B (*Lea2b*, *Hsp70b*), A-C (*Lea4*, *Hsp70c*), **A-B** (*Hsp20a*) **A-B** (*Hsp20b*).
